# White blood cells and coronary heart disease: A mendelian randomization study

**DOI:** 10.3389/fgene.2023.1127820

**Published:** 2023-02-07

**Authors:** Qiuping Zhao, Rongmei Liu, Hui Chen, Xiaomo Yang, Jiajia Dong, Minfu Bai

**Affiliations:** Fuwai Central China Cardiovascular Hospital, Heart Center of Henan Provincial People’s Hospital, Zhengzhou, China

**Keywords:** CHD, blood cell count, white blood cell count, mendelian randomization, instrumental variable (IV)

## Abstract

**Background:** The causal direction and magnitude of the associations between blood cell count and coronary heart disease (CHD) remain uncertain due to susceptibility of reverse causation and confounding. This study aimed to investigate the associations between blood cell count and CHD using Mendelian randomization (MR).

**Methods:** In this two-sample MR study, we identified independent blood cell count associated genetic variants from a genome-wide association studies (GWAS) among European ancestry individuals. Summary level data of CHD was obtained from a GWAS consisting of 547261 subjects. Methods of inverse variance weighted (IVW), Mendelian Randomization-Egger (MR-Egger), weighted median, and outlier test (MR-PRESSO) were conducted to investigate the associations between blood cell and CHD.

**Results:** Among all cardiovascular outcomes of interest, blood cell counts were only associated with CHD. Our findings indicated that white blood cell count and neutrophil cell count were significantly associated with increased risk of CHD [odds ratio (OR) = 1.07, 95% confidence interval (CI), 1.01–1.14; OR = 1.09, 1.02–1.16). However, there was no significant association between monocyte cell count, basophil cell count, lymphocyte cell count, eosinophil cell count, and CHD (*p* > 0.05). The results after excluding outliers were consistent with main results and the sensitivity analyses showed no evidence of pleiotropy (MR-Egger intercept, *p* > 0.05).

**Conclusion:** Our MR study suggested that greater white blood cell count and neutrophil cell count were associated with a higher risk of CHD. Future studies are still warranted to validate the results and investigate the mechanisms underlying these associations.

## Introduction

Coronary heart disease (CHD) is the leading cause of death all over the world, and the prevalence of cardiovascular diseases is increasing year by year (Heidenreich et al., 2011; Chua et al., 2022). The major sensitive diagnostic methods for CHD currently include electrocardiography, ultrasound, angiography and cardiac damage markers (Infante et al., 2017). However, these tests are limited by their expensive price, or the time required for testing, *etc.* Searching for new sensitive indicators and early detection of CHD can effectively guide management to prevent CHD related death (Gatto and Prati, 2020; Chow et al., 2021).

Coronary atherosclerosis is the main cause of CHD. It has been recognized that atherosclerosis is a complex chronic inflammatory disorder mediated through both adaptive and innate immunity (Taleb, 2016; Ruparelia and Choudhury, 2020). Previous studies demonstrated that proinflammatory cytokines (such as IL-1, IL-6, IL-1β and TNF α) participated in the development of CHD by endothelial damage, induction of monocyte-endothelial adhesion and subendothelial migration (Lusis, 2000; Ridker, 2019), and some large clinical trials have focused on the effects of targeted proinflammatory cytokines inhibition therapy on CHD (Ridker et al., 2017; Ridker et al., 2019). Pro-inflammatory cytokines are usually produced by immune cells such as macrophages, monocytes and dendritic cells. White blood cell (WBC), as the body’s predominant immune cells, have traditionally been recognized as markers of acute or chronic inflammation. Some observational studies have reported WBC and its subpopulations (lymphocytes, neutrophils, monocytes, eosinophils, and basophils) are associated with CHD (Wheeler et al., 2004; Madjid and Fatemi, 2013). However, the evidence is largely based on small sample studies, which are prone to systematic bias and do not support a causal association.

Mendelian randomization (MR) study is one in which genetic variants are used to investigate the causal relationship of a biomarker on risk of diseases (Smith and Ebrahim, 2003; Ference et al., 2012; Dale et al., 2017; Luo et al., 2021). Compared to the traditional observational studies that are susceptible to confounding bias and selection bias due to confounding factors, differences in study subject selection, *etc.* MR approach can be thought of as an analogy to a randomized controlled clinical trial (RCT) without the main biases of classical epidemiological studies (Bennett and Holmes, 2017). Recently, there have been a few studies using this genetic approach with large genome-wide association studies (GWAS) datasets to find causal effects of novel risk factors for CHD such as diet-derived circulating antioxidants, low-density lipoprotein cholesterol level, adiposity and body fat distribution and so on. (Ference et al., 2012; Dale et al., 2017; Luo et al., 2021). Therefore, there is an urgent need for MR studies to identify the causal relationships between WBC cell counts and CHD to help find targeted prevention and therapy for CHD.

In the present study, we employed MR analysis using data generated from large GWAS analysis to examine whether WBC and its major subpopulations may be causally linked to CHD.

## Methods

### Exposure and outcome GWAS data source

**FIGURE 1:**
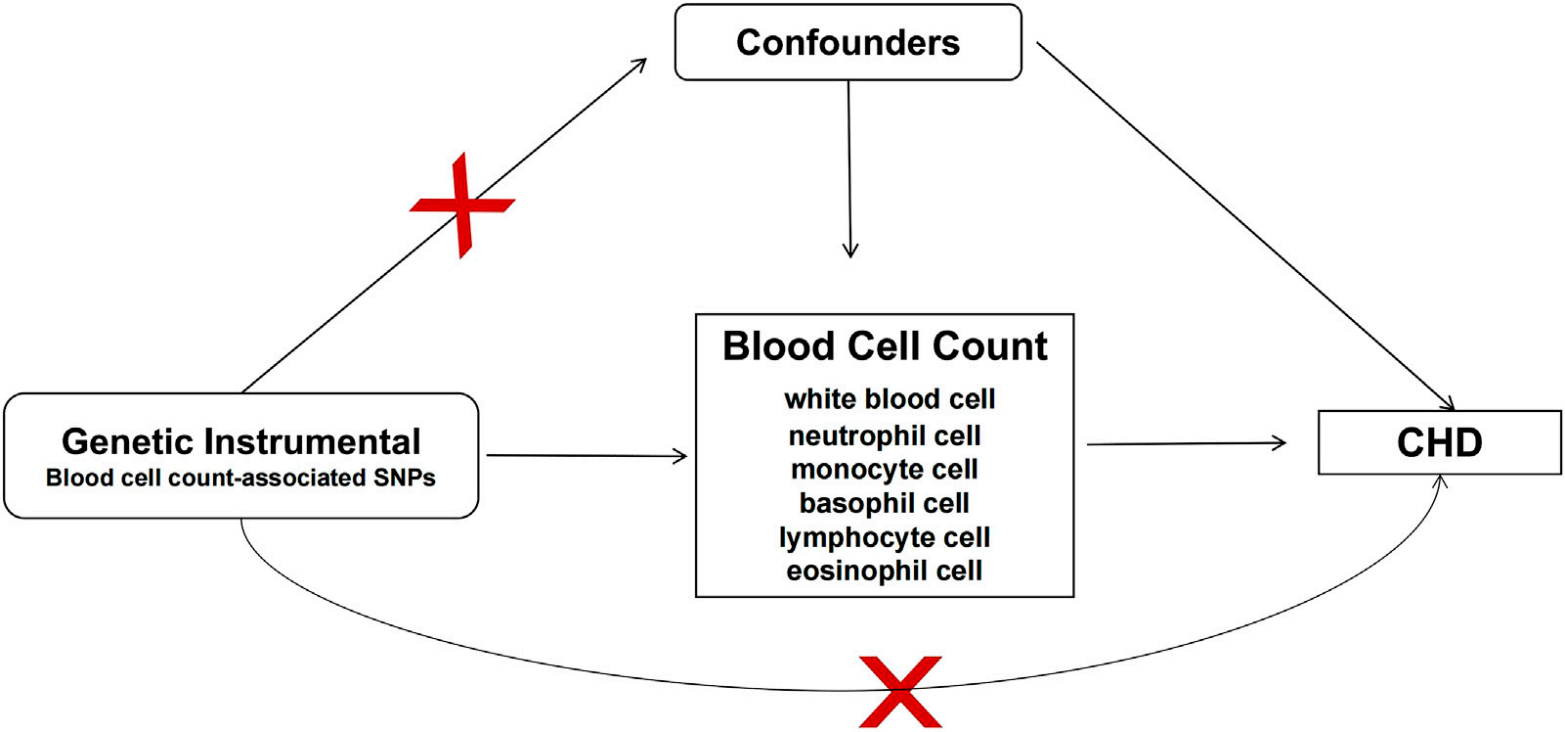
Study design of MR analysis. Conceptual schematic of the two-sample Mendelian randomization for the association between blood cell count and the risks of coronary heart disease. SNPs indicate single nucleotide polymorphisms; CHD, coronary heart disease.

The study design was shown in Figure 1. The summary GWAS data of blood cell counts were derived from the Blood Cell Consortium (BCX) meta-analysis, which includes data from European ancestry individuals (http://www.mhi-humangenetics.org/en/resources/). Blood cells in this study include total WBC, neutrophil cell, lymphocyte cell, monocyte cell, eosinophil cell, and basophil cell. Effect estimates of these Single-nucleotide polymorphism (SNPs) on the risk of CHD were evaluated with a GWAS, which involves 547,261 subjects (van der Harst and Verweij, 2018). Other cardiovascular disease (CVD) outcomes include heart failure (HF), ischemic stroke, and atrial fibrillation (AF). The summary-level data for HF was derived from a GWAS meta-analysis of HF comprising 47,309 cases and 930,014 controls (Shah et al., 2020). GWAS summary data of ischemic stroke was extracted from a meta-analysis of 12 individual GWAS studies with 29,633 (case/control = 10,307/19,326) participants (Malik et al., 2016). Data on AF from the GWAS meta-analysis included 537,409 participants, up to 55,114 cases and 482,295 controls (Roselli et al., 2018).

### Instrumental variable selection

The genetic instrument variables (IVs) for each blood cell type were identified using a statistically significant threshold with *p* < 5 × 10^−8^ and r^2^ < 0.001. To further identify independent IVs, we clumped the SNPs (Clumping r^2^ cutoff = 0.001, clumping window = 10 kb) based on the 1000 Genomes Project linkage disequilibrium (LD) reference panel, and the SNP with the lowest *p*-value is retained. For each of the SNP associated with exposure traits, we then extracted GWAS summary data of outcome. The maximum minor allele frequency (MAF) threshold for aligning palindromic SNPs was set at 0.3 and the LD r^2^ threshold for proxy SNPs from the 1,000 genomes Project was set at 0.8.

### Statistical analysis

The associations between blood cell count and CHD and other CVD outcomes were determined by two-sample MR analysis using inverse variance weighted (IVW), weighted median, and MR-Egger regression. The outcome was a binary variable, thus the results were shown as odds ratios (OR) and 95% confidence intervals (CI). The results of IVW were considered the primary causal effect estimates.

**FIGURE 2:**
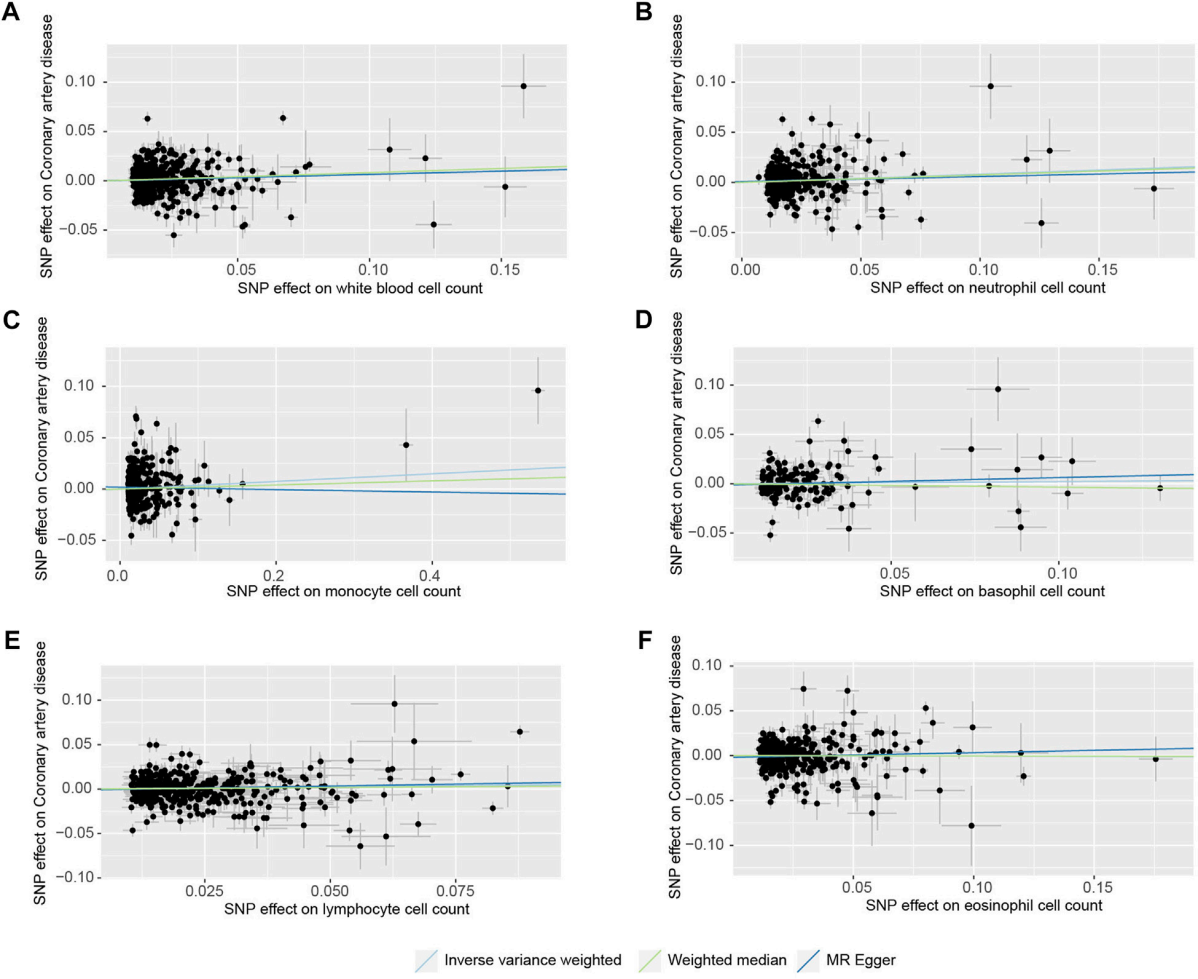
Scatter plots of the SNP effects on the blood cell count and coronary heart disease risk. **(A)**, white blood cell count; **(B)**, neutrophil cell count; **(C)**, monocyte cell count; **(D)**, basophil cell count; **(E)**, lymphocyte cell count; **(F)**, eosinophil cell count). Regression lines represent the causal effect of the blood cell count on coronary heart disease risk using IVW, MR-Egger, and Weighted median to estimate.

To account for the sensitivity of the results, we did several analyses. First, we performed a heterogeneity test and pleiotropy test to examine the reliability of MR estimates. We also depicted scatter plots for blood cell count and CHD to facilitate interpretation (Figure 2). Second, we also applied Mendelian Randomization Pleiotropy RESidual Sum and Outlier (MR-PRESSO) global test to further examine the reliability and identify horizontal pleiotropic outliers. If outliers are detected, we remove the outliers and repeated the analysis using the IVW method. All results were corrected for multiple testing using the false discovery rate (FDR) method and FDR q-value <0.05 were defined as statistically significant. After identifying the subtypes of white blood cell with an effect on CHD, we also conducted multivariate MR (MVMR) to estimate the effect of remaining white blood cell after adjusting for body mass index (BMI), current tobacco smoking, triglycerides (TG), or total cholesterol (TC). Finally, we performed wo-sample MR analysis to examine the effect of WBC in East Asian population. The detailed GWAS information was presented in Supplementary Table S13.

MR analysis was performed in R (version 4.0.3) with R packages “TwoSampleMR” and “MR-PRESSO”. *p*-value <0.05 was thought to be statistically significant.

## Result

### Character of SNP and participants for analysis

The characteristics of the populations included in the GWAS data of exposure (including white blood cell count, neutrophil cell count, monocyte cell count, basophil cell count, lymphocyte cell count, and eosinophil cell count) are shown in Table 1. Detailed information could be found in the original data (BCX). The sample size of GWAS data of outcome is 547,261, which comprised 122,733 cases and 424,528 controls. Table S1-S6 show the detailed information of each available SNPs used in the MR analysis.

**TABLE 1:** Characteristics of blood cell counts and coronary heart disease datasets.

Exposure	Consortium	Cases/Controls	Sample size	GWAS ID
white blood cell count	Blood Cell Consortium	—	563,946	ieu-b-30
neutrophil cell count	Blood Cell Consortium	—	563,946	ieu-b-34
monocyte cell count	Blood Cell Consortium	—	563,946	ieu-b-31
basophil cell count	Blood Cell Consortium	—	563,946	ieu-b-29
lymphocyte cell count	Blood Cell Consortium	—	563,946	ieu-b-32
eosinophil cell count	Blood Cell Consortium	—	563,946	ieu-b-33
Outcome	Consortium	Cases/Controls	Sample size	PMID
Coronary heart disease	—	122,733/424,528	547,261	29212778

GWAS, genome-wide association study.

### Causal effect of blood cell counts on coronary heart disease

The main results for CHD are presented in Table 2. For WBC count, a significant causal effect was observed (*p* < 0.05 in the 2 MR methods, including IVW and Weighted median). WBC count was associated with a higher risk of CHD (OR 1.07, 95% CI 1.01, 1.14). Besides, the OR and 95%CI of the neutrophil cell count were 1.09 (1.02, 1.16) for incident CHD risk. But there was no significant association between monocyte cell count, basophil cell count, lymphocyte cell count, eosinophil cell count, and CHD (OR, 1.04 95% CI 0.99, 1.08; OR 1.02, 95% CI 0.93, 1.12; OR 1.05, 95% CI 1.00, 1.11; OR 0.99, 95% CI 0.94, 1.05). Furthermore, the IVW results for WBC count and neutrophil cell count remained the multiple testing correction (FDR q < 0.05). There was no correlation between blood cell counts and other CVD outcomes, results shown in the Supplementary Table S7. We used a publicly available web tool (https://shiny.cnsgenomics.com/mRnd/) to evaluate the power of our study. For binary outcomes (CHD), after we inputed with a Type-I error rate α of 0.05 and the OR estimated by IVW method. The results of power calculations are shown in Supplementary Table S11.

**TABLE 2:** Two-sample Mendelian randomization estimations showing the effect of blood cell counts on the risk of coronary heart disease.

Blood cell counts	Method	OR (95%CI)	p-value	*P*h	Pintercept	q-value
white blood cell	MR Egger	1.07 (0.94.1.21)	0.31	3.55E-83	0.98	0.43
	Inverse variance weighted	1.07 (1.01.1.14)	0.03	6.24E-83		0.05
	Weighted median	1.09 (1.02.1.16)	0.02			0.07
neutrophil cell	MR Egger	1.05 (0.92.1.20)	0.45	3.88E-67	0.59	0.47
	Inverse variance weighted	1.09 (1.02.1.16)	0.01	5.00E-67		0.03
	Weighted median	1.08 (1.001.1.16)	0.05			0.09
monocyte cell	MR Egger	0.99 (0.92.1.06)	0.75	6.13E-59	0.09	0.60
	Inverse variance weighted	1.04 (0.99.1.08)	0.10	9.99E-60		0.09
	Weighted median	1.02 (0.97.1.07)	0.45			0.41
basophil cell	MR Egger	1.08 (0.89.1.30)	0.43	3.21E-30	0.50	0.46
	Inverse variance weighted	1.02 (0.93.1.12)	0.66	3.56E-30		0.40
	Weighted median	0.95 (0.82.1.11)	0.53			0.45
lymphocyte cell	MR Egger	1.09 (0.98.1.22)	0.12	4.05E-60	0.45	0.43
	Inverse variance weighted	1.05 (1.00.1.11)	0.07	4.11E-60		0.07
	Weighted median	1.04 (0.97.1.10)	0.26			0.31
eosinophil cell	MR Egger	1.05 (0.95.1.17)	0.32	3.33E-52	0.20	0.43
	Inverse variance weighted	0.99 (0.94.1.05)	0.84	1.39E-52		0.46
	Weighted median	1.00 (0.93.1.06)	0.91			0.43

MR, mendelian randomization; OR, odds ratio; CI, confidence interval.

### Sensitivity analysis validation

Several sensitivity analyses were conducted to estimate heterogeneity and horizontal pleiotropy in this MR analysis (Table 2; Supplementary Table S8). There was no horizontal pleiotropy in all results (P_intercept_ > 0.05). For heterogeneity, it was observed in all results of the heterogeneity test and MR-PRESSO global test (Ph < 0.05; Global Test-P < 0.05). According to the results of MR-PRESSO analysis, we detected some horizontal pleiotropic outliers in our analysis. After removing these outlier SNPs, the results showed consistency with the main results (Supplementary Table S8). WBC count and neutrophil cell count are still shown to be associated with CHD. The list of outliers for each exposure-outcome pair was presented in Table S9. The MVMR analysis, white blood cell counts still showed effects on CHD adjusted for BMI, current tobacco smoking, or TG. Neutrophil counts were also related with CHD after adjusting for BMI, current tobacco smoking, TC, or TG. All detailed MVMR results are shown in Supplementary Table S10. There was no significant association between white cell count and CHD in East Asian population, while the results all showed a positive trend (Supplementary Table S12).

## Discussion

In order to develop CHD prevention strategies, it is important to identify and compare causal risk factors in populations. In this study, we evaluated the causality of blood cell counts on CHD by MR analyses, we found that WBC count and the neutrophil cell count were associated with an increased risk of CHD, Additionally, no causal relationship was found between blood counts among other WBC types and CHD.

Currently, researchers have recognized that inflammation is a crucial factor in the pathophysiology process and mechanisms of clinical complications of CVD (Libby et al., 2016). Due to the inexpensive and widely-available, WBC and its subpopulation counts test have been the most commonly used test for inflammation. A small number of studies claim no association between WBC counts and CHD. In NHEFS cohort study, the association between neutrophil count and CHD disappeared after adjusting for age, gender, smoking and other risk factors (Gillum et al., 2005). However, more studies have demonstrated that WBC counts were associated with CVD and non-CVD mortality (Wheeler et al., 2004; Madjid and Fatemi, 2013; Welsh et al., 2018; Chan et al., 2022). Among the elderly Japanese-American male population cohort with 8 years of follow-up, high WBC count and neutrophil count were confirmed to be related to increased risk of incident CHD (Karino et al., 2015). Furthermore, recent data from UK biobank has shown that elevated neutrophil counts are particularly associated with a higher risk of CVD among the general population (Welsh et al., 2018). Our study is the first MR to explore the relationship between WBC counts and CHD, which effectively reduces the bias of observational line studies and clarifies the causal relationship between the two. Consistent with most previous studies, our results also show a strong association between WBC and neutrophil count with CHD. Further studies are needed to determine cut-points of WBC and its subpopulation and anti-inflammatory treatment protocols for CHD patients in the future.

According to research on the mechanism, leukocytes are engaged in the atherosclerosis process. Leukocytes can interact with structurally intact but dysfunctional arterial endothelium, and the development of a proinflammatory and prothrombotic environment following endothelial dysfunction leads to increased recruitment of leukocytes, lipids, smooth muscle cells, fibroblasts, and platelets to the arterial wall. Repeated cycles of this process lead to proliferation of the intimal lining of the vessel wall and formation of atherosclerotic plaques (Brydon et al., 2006). In addition, adhesion molecules allow leukocytes to attach to the endothelium monolayer when inflammation occurs. In the arterial plaque, pro-inflammatory cytokines stimulate leukocyte migration into the intima (Ilhan and Kalkanli, 2015). Oxidative processes play a significant part in the etiology of atherosclerosis. It has been hypothesized that white blood cells counts are implicated in the oxidative destruction of LDL that promotes atherosclerosis. (Wang et al., 2013).

Circulating neutrophils also contributed to pathophysiology of vascular disorders, particularly in the development of atherosclerosis. In mice, the researchers discovered that the size of the growing lesion was proportional to the number of circulating neutrophils (Silvestre-Roig et al., 2020). Complex structures called neutrophil extracellular traps (NETs), are released from suicide neutrophils. These traps contain proteins of nuclear, granular, and cell membrane origin as well as chromatin. Both human and mouse atherosclerotic lesions and artery thrombi have been shown to contain these reticular structures (Döring et al., 2017). When neutrophils come into contact with cholesterol, they generate NETs, and these NETs trigger the release of cytokines from macrophages, activating T helper 17 cells and so boosting immune cell recruitment to atherosclerotic plaques (Warnatsch et al., 2015).

Our study has some limitations. First, there was some horizontal pleiotropy in our study, which may result in a biased estimation of causal inference. However, we performed MR-PRESSO and the results remained consistent after removing the outlier SNPs. Secondly, the samples in our study were European and East Asian, which limits our results from being generalizable to other ethnic groups. The sample size of the East Asian GWAS data used here (*N* = 78,744) is much smaller than the sample size of the European population GWAS data (*N* = 563,946). Future research is needed to explore racial differences further. Despite relative weaknesses, our study also has several strengths. Our study used a large sample of GWAS data to explore the association between blood cell counts and CHD, which avoid confounding measurement error and residuals in observational studies. And we performed a series of sensitivity analyses to assess heterogeneity and pleiotropy, and we also conducted MR-PRESSO to remove the outlier SNPs. In addition, the present study conducted MR analysis, which uses genetic IVs analysis to model the randomization process that underlies causal inference in RCTs, thus was less susceptible to reverse causality bias. (Smith and Ebrahim, 2003).

## Conclusion

In conclusion, in this two-sample MR analysis, we demonstrated evidence for a potential causal link between WBC and neutrophil cells and CHD. In addition, causal relationships between blood cell counts among other WBC types and CHD were not found. In order to validate the results and investigate the mechanisms underlying these associations, future studies are still needed. The findings might help inform prevention strategies and suggest potential therapeutic targets for CHD.

## Data Availability

The original contributions presented in the study are included in the article/Supplementary Material, further inquiries can be directed to the corresponding author.

## Abbreviations

CHD, Coronary heart disease; WBC, White blood cell; MR, Mendelian randomization; RCT, randomized controlled clinical trial; GWAS, genome-wide association studies; BCX, Blood Cell Consortium; MR-PRESSO, MR-Pleiotropy Residual Sum and Outlier; OR, odds ratio; IVW, inverse-variance weighted; CI, confidence intervals; IV, instrumental variable; SNP, Single-nucleotide polymorphism; LD, linkage disequilibrium; MVMR, multivariate Mendelian randomization; BMI, body mass index; TC, triglycerides; TG, total cholesterol; NETs, neutrophil extracellular traps; MAF, minor allele frequency.
